# Frequency of Anemia in the Patients of a Family Health Team in Dourados, Mato Grosso do Sul, Brazil

**DOI:** 10.1155/2011/836184

**Published:** 2011-08-13

**Authors:** Tatiana Lachi, Cássia Barbosa Reis

**Affiliations:** ^1^Universidade Federal da Grande Dourados, Rua João Rosa Goes no. 1761, Vila Progresso, Caixa Postal 322, 79825-070 Dourados, MS, Brazil; ^2^Universidade Estadual de Mato Grosso do Sul, Cidade Universitária de Dourados, Caixa Postal 351, 79804-970 Dourados, MS, Brazil

## Abstract

Anemia is a clinical manifestation with high prevalence in the world, reaching about 30% of the total inhabitants of the planet. It is responsible for a great reduction in quality of life of affected people. The present paper aimed to obtain the frequency of anemia in the blood counts of a sample from the population of registered patients of a Family Health Team in Dourados, MS, Brazil. 462 patients, who had blood counts done from February 2008 to March 2009, were included in this research. The frequency of anemia was 22.7% in the studied population, including all age groups.

## 1. Introduction

Anemia is a common finding in the world population, affecting both developing countries and industrialized nations. It reaches about 2 billion people worldwide, or about 30% of the total inhabitants of the planet, according to data from the World Health Organization-WHO. Because of such magnitude, it is considered a public health problem. It can greatly affect a person's life, especially in its more severe forms. Anemia causes tiredness, weakness, malaise, and making routine tasks more difficult and painful [1]. The affected person may be drowsy, with less resistance to physical activities and deterioration in cognitive function and immunity [2]. A drop in libido may occur [1]. Thus, quality of life can be seriously injured. Nutritional lack of iron leads to the most frequent type of anemia, iron deficiency anemia. Even motor development in children can be disturbed by iron deficiency [3–5], but this nutritional privation has easy and inexpensive care and prophylaxis. Thus, government or personal expenses to reverse its effects or avoid iron deficiency anemia are not large and can bring great improvements to the lives of individuals. In the staff's routine of the Family Health Strategy studied in Dourados, MS, Brazil, it can be seen that anemia affects a lot of patients. Therefore, a study about the frequency of that clinical manifestation can provide great information for the planning of prevention and treatment. The aim of this research was to obtain the frequency of anemia in blood counts of those patients.

## 2. Methods

A quantitative and descriptive study, with a retrospective component, using secondary data and blood counts from the medical records of patients subjected to research, was conducted in the city of Dourados, MS, Brazil. Patients ascribed to the Family Health Strategy studied totalize 4,244 people and a purposive sample of 462 patients from that population was used. The sample consists of patients who attended the Health Unit and had blood counts done during the period from February 2008 to March 2009. Pregnant patients were excluded. Data were collected through forms made by the author containing information on sex, age, and hematologic condition of the research subjects. Results were organized into charts and tables with descriptive statistical analysis through Software Excel 2007 and Epi-Info 3.3.2. This research was authorized by Committee of Stage, Practical Class, Research and/or Final Course Work from the Municipal Health Department–CEPET, and it was also approved by Ethics Committee on Human Research from Federal University of Mato Grosso do Sul-UFMS, according to Ordinance MS 196/96, protocol number 1497. Rules for use of information from patients' records are mentioned in items III.3.i and III.3.t of Resolution CNS 196/96 and they were followed. The present research did not entail risks to patients, since the blood samples had already been collected. It benefits population, since, from the determination of frequency of anemia, prevention and treatment actions can be made.

## 3. Results

### 3.1. General Frequency of Anemia

The frequency of anemia obtained in the studied sample was 22,7%, including all age groups.

### 3.2. Frequency of Anemia by Age

**Figure 1:**
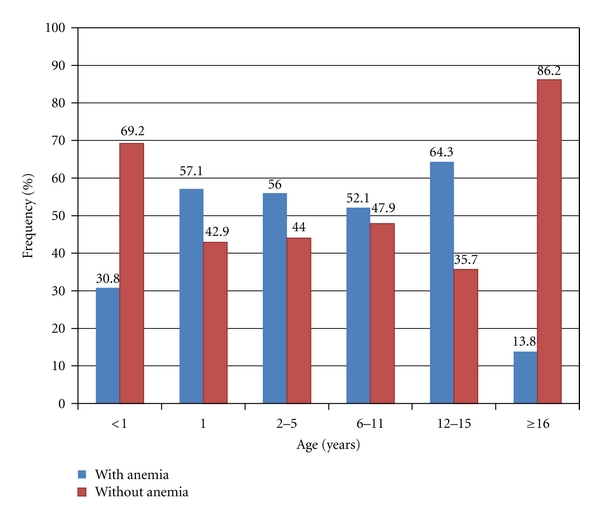
Frequency of anemia in users of the Family Health Strategy studied, according to age range, from February 2008 to March 2009, Dourados, MS, Brazil.

The frequency of anemia by age in the studied sample is shown in Figure 1. Among the teenagers from 12 to 15 years old, 64.3% were anemic. Among children aged 1 year, this occurrence was 57.1%, and in those aged 2–5 years, it was 56%. Anemia was found in 52.1% of children from 6 to 12 years. In children under 1 year in the sample, 30.8% were anemic, while 13.8% of people aged 16 years or more showed the same clinical manifestation at the time of collecting their blood counts. The oldest patient was 92 years old.

 Tables 1 and 2 show the values of hemoglobin and the hematocrit found in the sample studied in relation to age range.  Table 3 correlates the MCV (Mean Corpuscular Volume) values in relation to age range. Among children younger than 1 year, the most common hemoglobin values varied from 11.0 to 12.4 g/dL (or g%), the range in which 53.8% of those patients were in (Table 1). The most frequent hematocrit values in the same patients were between 36 and 40.9%, a range spanning 38.5% of those children (Table 2). 

In patients aged from 1 to 15 years old and older than 15 years old, the most common hemoglobin values were between 12.5 and 13.9 g/dL, observed in 41.1% and 38.1% of those populations, respectively (Table 1), while their most frequent hematocrit ranged between 36 and 40.9%, values found in 64.2% and 41% of those populations, respectively (Table 2). The hemoglobin and hematocrit found have meaning only if analyzed at the individual level, since they vary according to each age. Such analysis was subject of Figure 1. 

Table 3 shows that the most frequent values of Mean Corpuscular Volume (MCV) among children under 1 year old varied from 80 to 84.9 *μ* ^3^, which are the values found in 46.2% of those patients, while both the population aged from 1 to 15 years old and aged over 15 years old showed most frequent MCV values between 85 and 89.9 *μ* ^3^. Those values were observed in 52.6% of patients aged from 1 to 15 years old and in 50.0% of patients over 15 years old.

The Mean Corpuscular Hemoglobin (MCH) values in 61.6% of patients younger than 1 year old were between 25 and 28.9 ng—30.8% of them with values between 25 and 26.9 ng and other 30.8% with values between 27 and 28.9 ng. It was observed that 45.3% of patients aged from 1 to 15 years old had values of MCH between 27 and 28.9 ng, while 71.8% of patients over age 15 had MCH values between 29 and 30.9 ng (Table 4).

Table 5 discriminates the values of Mean Corpuscular Hemoglobin Concentration (MCHC) that were found in each age. It is observed that in all age groups the most frequent MCHC values ranged from 32 to 33.9%. Such values were found in 69.2% of patients younger than 1 year old, 83.2% of patients aged from 1 to 15 years old and 91.5% of patients over 15 years old. 

Children under 1 year old have different reference values for hemoglobin, hematocrit, MCV, MCH, and MCHC, according to age. Figure 1 was constructed by analyzing each child individually. In tables, they were grouped into denomination “younger than 1 year old” to easy viewing. 

Table 6 shows the reference values for each parameter (erythrocytes, hemoglobin, hematocrit, MCV, MCH, and MCHC) according to age.

## 4. Discussion

The anemia frequency found in nearly 23% in population is high and it approaches those values found in the world: about 30% of its population were affected by anemia [6]. Such clinical manifestation can affect quality of life by decreasing exercise tolerance, impairing learning ability, affecting libido, causing fatigue, discomfort, and reducing appetite among other changes. Thus, over 20% of people who attended the Family Health Strategy team and who were included in this research face damage in their ability to perform daily activities, because of a blood disorder that can be easily avoided and also easily reversed once installed. 

The presence of infectious processes in children and adolescents when blood counts were collected may have lead to a bias in the results, possibly overestimating frequency of real anemia, since a transient anemia can be found during an infectious process.

Anemias do not spare more affluent social classes. And the high number of anemia cases found in the study population proves what is observed throughout the country: anemias are the most prominent endemic deficiency, overcoming the lack of iodine (which is currently under control), vitamin A, and even protein-calorie malnutrition [7].

After treatment in the population of our study, based on ferrous sulfate, it was possible to reverse most cases of anemia found. So iron deficiency anemia is supposed to be the most prevalent type of this clinical manifestation in those people. The values of Mean Corpuscular Volume, Mean Corpuscular Hemoglobin, and Mean Corpuscular Hemoglobin Concentration are needed if analyzed individually correlated with the values of hemoglobin (Hb) and hematocrit (Ht) for each patient studied and combined with additional laboratory tests, not available in records analyzed. Such analysis may be the object of further researches.

It can be seen in this study that the sample has prevalence of women: 62.6% of patients (95% CI 58.0 to 67.0). This is due to historical characterization that women are the majority of people who attend health services. 

The values of hemoglobin and hematocrit found in patients with anemia in the study population characterize that clinical manifestation as mild in most cases and in all ages. There were few cases of moderate to severe anemia, which responded promptly to therapy with oral or parenteral replacement of iron, being characterized, although the lack of additional laboratory tests available, as iron deficiency anemia, because of its rapid response to treatment. The WHO defines mild, moderate, and severe anemia according to hemoglobin values. Mild anemia is characterized by hemoglobin values between 11.0 and 11.9 mg/dL in children and adult women and between 12 and 12.9 mg/dL in adult men. Moderate anemia occurs when hemoglobin values vary from 8.0 to 10.9 in children and adult women, and from 9.0 to 11.9 in adult men, and severe anemia has hemoglobin values from 5.0 to 7.9 in children and adult women and from 6.0 to 8.9 in adult men.

The findings of the present research are similar to those observed throughout Brazil, where anemia reaches about 50% of children between 6 months and 2 years old [6] and between 40 and 50% of children under 5 years old [7]. 

The iron fortification in widely consumed foods in each population was sustained as a measure of greater scope to try reducing such a high prevalence of anemia. Several studies show that food fortification can reduce nutritional deficiencies [8]. The supply of iron supplementation for the most vulnerable population groups (such as children, women at reproductive age, and pregnant women), food fortification, educational measures, and information to the population are strategies for combating anemia. If added together, they can be effective in fighting this serious public health problem all over the world [7].

## 5. Conclusions

The high frequency of anemia in the study population follows what can be seen worldwide. This clinical manifestation does not discriminate between rich or poor countries and affects a great number of people in several nations. A person's life can be greatly disturbed by its symptoms, such as tiredness, lower immunity, impairment in the growth of children, in cognitive functions, and libido [1, 2]. Lack of iron is a very common nutritional deficiency and it is responsible for the most frequent type of anemia in the world, the iron deficiency anemia [5]. The treatment in those cases is simple and inexpensive, and so is its prevention. Avoiding or treating anemia can contribute to a higher quality of life for people.

## Figures and Tables

**Table 1:** Hemoglobin values according to age range, Dourados, MS, Brazil, 2009.

Hemoglobin values (g/dL or g%)*	Younger than 1 year old		From 1 to 15 years old		Older than 15 years old	
	*N *	%	*N *	%	*N *	%
6.5–7.9	1	7.7	—	—	1	0.3
8.0–9.4	2	15.4	2	2.1	2	0.6
9.5–10.9	3	23.1	14	14.7	12	3.4
11.0–12.4	7	53.8	36	37.9	63	17.8
12.5–13.9	—	—	39	41.1	135	38.1
14.0–15.4	—	—	3	3.2	115	32.5
15.5–16.9	—	—	1	1.1	24	6.8
17.0–18.4	—	—	—	—	2	0.6

Total	13	100	95	100	354	100

*g/dL: grams per deciliter of blood.

**Table 2:** Hematocrit values according to age range, Dourados, MS, Brazil, 2009.

Hematocrit values (%)	Younger than 1 year old		From 1 to 15 years old		Older than 15 years old	
	*N *	%	*N *	%	*N *	%
21–25.9	1	7.7	—	—	3	0.8
26–30.9	3	23.1	3	3.2	4	1.1
31–35.9	4	30.8	24	25.3	28	7.9
36–40.9	5	38.5	61	64.2	145	41.0
41–45.9	—	—	6	6.3	135	38.1
46–50.9	—	—	—	—	37	10.5
51–55.9	—	—	1	1.1	2	0.6

Total	13	100	95	100	354	100

**Table 3:** Mean Corpuscular Volume (MCV) values according to age range, Dourados/MS, Brazil, 2009.

MCV values (*μ* ^3^)	Younger than 1 year old		From 1 to 15 years old		Older than 15 years old	
	*N *	%	*N *	%	*N *	%
50–55	—	—	—	—	1	0.3
55–60	—	—	—	—	—	—
60–65	1	7.7	1	1.1	1	0.3
65–70	—	—	1	1.1	3	0.8
70–75	3	23.1	4	4.2	1	0.3
75–80	2	15.4	11	11.6	2	0.6
80–85	6	46.2	17	17.9	18	5.1
85–90	—	—	50	52.6	177	50.0
90–95	1	7.7	11	11.6	149	42.1
95–100	—	—	—	—	2	0.6

Total	13	100	95	100	354	100

**Table 4:** Mean Corpuscular Hemoglobin (MCH) values according to age range, Dourados/MS, Brazil, 2009.

MCH Values (ng)	Younger than 1 year old		From 1 to 15 years old		Older than 15 years old	
	*N *	%	*N *	%	*N *	%
15–16.9	—	—	1	1.1	1	0.3
17–18.9	—	—	—	—	—	—
19–20.9	1	7.7	—	—	1	0.3
21–22.9	1	7.7	3	3.2	3	0.8
23–24.9	3	23.1	3	3.2	1	0.3
25–26.9	4	30.8	14	14.7	5	1.4
27–28.9	4	30.8	43	45.3	65	18.4
29–30.9	—	—	30	31.6	254	71.8
31–32.9	—	—	1	1.1	21	5.9
33–34.9	—	—	—	—	3	0.8

Total	13	100	95	100	354	100

**Table 5:** Mean Corpuscular Hemoglobin Concentration (MCHC) values according to age range, Dourados, MS, 2009.

MCHC values (%)	Younger than 1 year old		From 1 to 15 years old		Older than 15 years old	
	*N *	%	*N *	%	*N *	%
26–27.9	—	—	1	1.1	1	0.3
28–29.9	—	—	—	—	2	0.6
30–31.9	3	23.1	7	7.4	9	2.5
32–33.9	9	69.2	79	83.2	324	91.5
34–35.9	1	7.7	8	8.4	17	4.8
36–37.9	—	—	—	—	1	0.3

Total	13	100	95	100	354	100

**Table 6:** Reference values for erythrocytes, hemoglobin, hematocrit, MCV, MCH, and MCHC according to age. Source: Clinical Laboratory, University Hospital of Dourados, MS, Brazil. Brazilian Association of Hematology and Hemotherapy—(ABHH).

Age	Hemoglobin (g/dL)	Hematocrit (%)	MCV (u^3^)	MCH (ng)	MCHC (%)
5 days	19-20	60–65	92–99	30–40	32–36
14 days	12.5–15.7	42–48	60–70	25–30	27–34
2 months	14–19	40–50	90–97	28–33	31–34
6 months	9.5–13.5	29–41	74–99	25–35	30–36
12 months	11.8–12.6	35–40	75–80	25–30	33–37
4 years	12.3–13.8	35–40	75–80	28–30	33–37
8 years	12.3–14	38–42	75–85	28–30	33–37
14 years	13.5–14.5	40–50	82–92	28–30	32–36
Adult men	14–16	40–54	82–92	27–32	32–36
Adult women	11–14	37–47	82–92	27–32	32–36
